# Challenges of Immune Response Diversity in the Human Population Concerning New Tuberculosis Diagnostics, Therapies, and Vaccines

**DOI:** 10.3389/fcimb.2020.00139

**Published:** 2020-04-08

**Authors:** Abul K. Azad, Christopher Lloyd, Wolfgang Sadee, Larry S. Schlesinger

**Affiliations:** ^1^Host-Pathogen Interaction Program, Texas Biomedical Research Institute, San Antonio, TX, United States; ^2^Department of Cancer Biology and Genetics, Center for Pharmacogenomics, College of Medicine, The Ohio State University, Columbus, OH, United States

**Keywords:** tuberculosis, immunity, diagnostics, therapy, vaccine, genetics, genetic diversity

## Abstract

Universal approaches to the prevention and treatment of human diseases fail to take into account profound immune diversity resulting from genetic variations across populations. Personalized or precision medicine takes into account individual lifestyle, environment, and biology (genetics and immune status) and is being adopted in several disease intervention strategies such as cancer and heart disease. However, its application in infectious diseases, particularly global diseases such as tuberculosis (TB), is far more complex and in a state of infancy. Here, we discuss the impact of human genetic variations on immune responses and how they relate to failures seen in current TB diagnostic, therapy, and vaccine approaches across populations. We offer our perspective on the challenges and potential for more refined approaches going forward.

## The Close Link Between Human Evolutionary Biology and Immune Diversity

Human evolutionary biology is thought to have a pronounced influence on genetic diversity, particularly in regards to the immune system. Disease-risk alleles that are predominantly deleterious by loss of function represent a subset of global human genetic diversity. Their occurrence, frequency, and population distribution are shaped by evolutionary forces such as purifying selection, genetic drift, and migration (Tishkoff and Verrelli, 2003; Quintana-Murci, 2016). On the other hand, genetic variants that convey protection and resilience can be under positive selection, reaching high allele frequencies, observed in genes affecting immunity and host defense, under the enduring selection pressure of microbes.

Large inter-individual variation observed in human immune responses has been attributed to genetic factors (Azad et al., 2012; Sanz et al., 2018; Scepanovic et al., 2018) and environmental influence (Macgillivray and Kollmann, 2014), subject to both human adaptation and rapid evolution of the pathogens (Kodaman et al., 2014). Admixture with ancient hominins introduced advantageous immune variants into the modern human population (Racimo et al., 2015), and it is unlikely that the population distribution of common susceptibility alleles results from neutral processes alone (Corona et al., 2013; Quintana-Murci, 2016). The innate immune system is the critical primary target for protection against infections, with a large portion of the human population exposed to a variety of infections, one prominent one being *Mycobacterium tuberculosis* (*M.tb*). However, ramping up the innate immune system—necessary under increasingly crowded living conditions—engenders susceptibility to autoimmune diseases (Waldner, 2009; Azad et al., 2012). As a result, genetic variants in innate immune genes often accumulate to high allele frequency until the negative and positive effects cancel each other. This type of genetic variant under balancing selection is largely regulatory, requiring distinct methods for discovery compared to variants that change the amino acid sequence. Because multiple pathways can lead to resistance against various infectious agents and their subspecies, we expect pronounced differences in genetic adaptation between populations, and, in parallel, genetic risk of diseases such as type 2 diabetes, biliary liver cirrhosis, psoriasis, inflammatory bowel disease, and systemic lupus erythematosus (Corona et al., 2013). These relationships lead to pervasive overlap between positively selected genomic regions and genes associated with traits or diseases in genome-wide association studies (GWAS) that comprise multiple regions associated with infectious and autoimmune disease risks, including tuberculosis (TB) and leprosy (Grossman et al., 2013). Medications that target chemokines and cytokines—principal actors of the innate immune response—hence carry warning labels not to be used when an infection such as TB is detected, highlighting the nexus between autoimmune disease and infection. In the context of how human evolution has acted upon immune phenotypes and diversity, it is important to consider that adapted immune traits can be transmitted across generations not only through genetics but also *via* environmental and cultural factors (Danchin et al., 2011).

## Importance of Inter-Individual Human Immune Response Variation in Infectious Diseases Such as TB

When humans are exposed to pathogenic microbes, immune responses are initiated to protect the host, and these responses are highly variable among humans, even between healthy individuals within and across populations (Scepanovic et al., 2018). In the case of TB, *M.tb* infection is followed by a spectrum of responses and outcomes (Lin and Flynn, 2018). Very few infected individuals proceed to develop clinical disease. Instead, the majority of those infected maintain latent TB infection (LTBI), with only 5–10% later developing clinical disease, usually within 2 years of infection (Dodd and Schlesinger, 2017; Behr et al., 2018).

Human TB cell-based experimentation studies and vaccine trials highlight the significant hurdle of substantial human-to-human variation in cellular and immunological responses to antigen stimulation (Azad et al., 2012; Tameris et al., 2013; Zumla et al., 2016; Fava and Schurr, 2017). We posit that knowing the root causes of differential human immune responses will enable better design of effective therapies and vaccines that encompass the majority of recipients. While several factors contribute to natural differences in human immunity to disease, this PERSPECTIVE article focuses mainly on host genetic factors as one of the main drivers for outcomes of infectious diseases, specifically TB (Abel et al., 2014). In the current state, the BCG vaccine for TB elicits highly variable protection in different human populations, and anti-TB drugs might work well in one person but be less effective or cause serious side effects in another (Bloom et al., 2017). Therefore, knowing the underlying relationship between genetics and biology will enable us to identify those individuals who will benefit maximally from designed therapies and vaccines.

## Global Database on TB Incidence at a Glance

According to recent WHO report (2019), an estimated 10.0 million (range, 9.0–11.1 million) people fell ill with TB in 2018 (WHO, 2019). Globally, the average rate of decline in the TB incidence rate was 1.6% per year in the period 2000–2018, and 2.0% between 2017 and 2018. The global reduction in the number of TB deaths between 2015 and 2018 was 11%. Co-infection (for example, with HIV) bears influence on TB mortality. There were 1.2 million TB deaths among HIV-negative people globally in 2018, but an additional 251,000 deaths were reported among HIV-positive people. Since 8.6% of all TB cases were also living with HIV, it appears that co-infection with HIV increases the death rate. Geographically, most TB incidences in 2018 were in the WHO regions of developing countries of South-East Asia (44%), Africa (24%) and the Western Pacific (18%), with smaller shares in most developed countries of the Eastern Mediterranean (8%), the Americas (3%) and Europe (3%). In 2017, 69% of international donor funding for TB was provided by the Global Fund. The US government was the second largest donor, which contributed US$ 249 million (23% of the global total) via the Global Fund (WHO, 2019).

## Challenges of Universal Medicine and the Prospect of More Personalized Treatments For TB

*M.tb* infection leads to significant up-regulation of many immune and metabolic response genes (Barreiro et al., 2012). However, if the individual carries functional genetic variants [most commonly single nucleotide polymorphisms (SNPs)] in key immune or metabolic response genes, primary immune responses that defend against *M.tb* infection can be rendered more or less robust (Barreiro et al., 2012). Likewise, if genetic variants significantly alter drug metabolism, a standard drug formulation could fail or cause side effects in carriers (Ahmed et al., 2016). In the ideal situation, each individual's personal set of immune and metabolic characteristics should be considered to provide the most effective treatment for TB and other infectious diseases. This challenge is already being addressed in deciding on cancer immunotherapy regimens (Liu and Guo, 2018). Serious problems may arise for immune-variant individuals when universal medicine, the provision of identical forms of treatment to all individuals who present with the same disease, is exercised; yet, our ability to predict immune competence with respect to infectious diseases is quite limited. Heterogeneity between ethnic groups contributes to the challenge of developing biomarker panels guiding therapy. For example, 16–21% of genes involved in generating Toll-like receptor (TLR) ligands and other immune responses differ between African and European subjects (Sanz et al., 2018). Such findings suggest that immunity across different populations will differ enough to render treatments that are effective in one region ineffective in others, further illustrating the need to optimize treatments to personal characteristics. Similarly, frequent variants exist in genes encoding drug-metabolizing enzymes, for example, N-acetyltransferase 2 (NAT2), which plays a role in the metabolism of the anti-TB drug isoniazid (INH) (Klein et al., 2016) (see below). To date, genetic predictors are not being used in the clinic for diagnostic and therapeutic purposes in infectious diseases, which is in stark contrast to their pervasive use in cancer and other diseases (Thanassoulis and Vasan, 2010; Tian et al., 2012; Verma, 2012; Demkow and Wolanczyk, 2017; Giudicessi et al., 2017).

For these reasons, we argue that new treatments and vaccines designed on a more personal basis in infectious diseases including TB should be considered going forward, so-called personalized or precision medicine. Improved genetic sequencing technologies, functional genomics strategies, and systems biology approaches developed in recent years make this feasible to consider (Poland et al., 2013; Jensen and Van Hal, 2017; Ladner et al., 2019). Such approaches have allowed for fast and reliable sequencing of both human and pathogen genomes, providing increased knowledge of host-pathogen interactions, particularly the underlying mechanisms for disease pathogenesis, which can be used as the foundation for developing more personalized treatments, particularly relevant when considering host-directed therapies (Simmons et al., 2018). There has been a recent discussion on the development of precision medicine for drug-resistant TB (Cox et al., 2018; Groschel et al., 2018; Mahomed et al., 2019). However, interpretation of human genomics data in the context of susceptibility to infections is currently far from sufficient to develop clinical decision tools. In part, this shortcoming may have arisen from the emphasis of research on those of European descent (Popejoy and Fullerton, 2016), biasing conclusions and missing opportunities to learn from the global variation of the human immune system.

Although the concept and application of personalized medicine are attractive and hold promise for avoiding unnecessary and inappropriate treatment options, there are indeed drawbacks. Apart from ethical issues, the introduction of personalized medicine will be difficult to institute in endemic regions, especially in developing countries, where poverty, lawlessness, and internal strife exist. According to many critics, the application of personalized medicine theory in developing countries is considered uncertain or impossible (So and Joly, 2013; Gameiro et al., 2018). In addition to the limited global financial support for the development of new costly medicine, collected genomic data remain insufficient for many ethnic groups. Current GWAS go a long way to cover ethnic diversity, but often lack sufficient size to advance the field substantially. While it can be reasonably argued that personalized medicine cannot yet be broadly applied to the individual, we submit that more emphasis should be placed on initiatives to progress precision medicine principles on a regionalized scale, covering the majority of ethnic populations, and leading to novel insights into relevant disease risk pathways in those populations.

## Human Genetic Polymorphisms in TB-Associated Innate Immune Genes, Gwas, Functional Genomics, and Pharmacogenomics

Functional variants in host innate immune genes are frequent and include both gain- and loss-of-function mutations under strong selection pressure. Key genes affected are likely to be shared across all ethnicities. However, we can further expect that multiple variants have arisen in each gene locus, that interact with each other, with substantially different allele frequencies between ethnic groups—a challenge for geneticists assessing functional consequences. Host genetic risk in TB also depends on the mycobacterial genetic background, highlighting the importance of studying the interaction between host and *M.tb* genomes (Omae et al., 2017). TB-associated innate immune genes encode pattern recognition receptors, inflammatory factors, nuclear receptors, surfactant proteins, oxidative response proteins, cytokines, chemokines, etc., (Azad et al., 2012; Fol et al., 2015). A majority of TB-associated SNPs have been derived from GWAS, taking advantage of massive genomics data sets across populations, leading to inferences of candidate genes contributing to human immune variation and susceptibility or resistance to diseases in general (Uren et al., 2017). However, GWAS-significant SNPs mostly do not reveal the underlying mechanisms of a potential functional variant or even the causative genes, as regulatory variants can reside at a long distance from their target genes, acting by chromatin looping mechanisms (Sadee et al., 2014; Gallagher and Chen-Plotkin, 2018). Therefore, GWAS-significant SNPs are often only marker SNPs that vary greatly in frequency between ethnic groups. Moreover, since TB is a complex multi-genic disorder, each genetic variant alone contributes but a small fraction of overall risk which can, in part, be addressed with polygenic risk scores reflecting all marker SNPs (Wray et al., 2013). However, this approach neglects dynamic interactions between genes and pathways and with the environment. Much larger datasets are needed to begin to address these crucial questions (currently under development in multiple countries) making it difficult at the current time to identify any single SNP or combination of SNPs affecting gene product(s) that can be targeted for therapy or vaccine development (Jin et al., 2019; Luo et al., 2019). On the other hand, a rapidly expanding source of large functional genomic databases is now publicly available enabling identification of causal variants and genes, using expression quantitative trait locus analysis presented in GTEx (Consortium, 2013), and the Encyclopedia of DNA Elements (ENCODE) (Consortium, 2012), followed by experimental validation using molecular biology tools. The field of pharmacogenomics has embraced functional genomics platforms for some time, identifying causal functional variants that alter drug metabolism, thereby applying personalized medicine concepts to individualizing drug treatment (Potamias et al., 2014) (see below).

Current GWAS for TB are still limited by sample size, ethnic stratification of the sample population, and difficulties in acquiring accurate medical history records such as information about past exposure to disease and treatment (Barreiro et al., 2012). As a result of hidden population stratification, GWAS-hit SNPs are often spurious, requiring mandatory replication in the same ethnic population and across ethnic groups—where SNPs may vary, but candidate genes may overlap. A particular hurdle in GWAS is a large number of variants tested, up to 4 million per chip plus imputations, each to be considered a separate hypothesis—hence, genome-wide significance requires a *p*-value as low as 10^−8^ under Bonferroni correction. Clearly, many valid functional variants remain hidden using this conservative cutoff. Therefore, one can use prior knowledge to focus on a genomic region around a known strong candidate immune gene, greatly reducing the number of SNPs tested and hence allowing for a higher *p*-value, under the assumption that a causative variant is often located near its immune target gene (e.g., within 200-kb of the gene's transcription starting site in either direction) (Barreiro et al., 2012). To search for more than one functional variant per gene locus, one needs to address the linkage disequilibrium (LD) structure (as available in the 1,000 genomes database) to determine which LD block carries independently active variants (Uren et al., 2017) and how these could interact with each other (Lee et al., 2018; Joiret et al., 2019).

## Immune Diversity and the Challenge of Global TB Diagnostics and Vaccines

Variability in immune responses can contribute to the difficulty of TB diagnosis. Currently, the tuberculin skin test and blood IFNγ release assay are used to assess TB infection (Sharma et al., 2017). Due to the different mechanisms by which these tests operate, results do not always agree (Gallant et al., 2010). In addition, the presence of immune-related genetic variants likely affects test results of immune responses as indicators of *M.tb* infection. A report of the influence of genetic variance and shared environment on the production of IFNγ is an example in this context (Tao et al., 2013).

Genetic variability between individuals also poses a challenge to developing a universal TB vaccine (Gong et al., 2018). Substantial variability in response to the current BCG vaccine is attributed to individual differences in age, sex, dosage, method of vaccination, and nutritional status, among others (Castiblanco and Anaya, 2015; Newport, 2015). Genetic variants that influence the expression of TLRs, HLAs, and cytokines and their receptors can induce differential host responses to the BCG vaccine (Newport, 2015). For example, IFNγ responses in Gambian neonates after BCG vaccination demonstrate a bell-shaped curve distribution, indicating that the vaccine does not induce immunity across all individuals in a population (Newport, 2015). A twin study in Gambian infants indicates that IFNγ and IL-13 responses to tetanus, pertussis, and some BCG vaccine antigens are 39–65% heritable (Newport et al., 2004), supporting the view that immune response to vaccines is strongly influenced by genetics and that the genetic profile of individuals should be taken into account when developing effective TB vaccines. This critical issue compels a robust effort to understand the underlying biology and genetics, which are currently insufficiently resolved to guide vaccine optimization.

Current TB vaccine design strategies generally target enhanced cell-mediated immunity, whereas antibody-based approaches have been largely dismissed until recently (Achkar and Casadevall, 2013; Cywes-Bentley et al., 2018; Kawahara et al., 2019; Tanner et al., 2019). Recent evidence also supports the contribution of trained innate immunity to vaccine protection (Khader et al., 2019; Koeken et al., 2019). Since infants and the elderly are at high risk of infectious diseases, researchers are beginning to focus on understanding age-specific immunity to design workable vaccines for those age-groups (Keener, 2019). Future vaccine approaches will need to consider the different immune mechanisms in these contexts but will also need to consider strategies to identify subsets of individuals, populations, or regions in order to determine the best vaccine type or adjuvant combination to administer. Such efforts will also further clarify the degree of susceptibility or resistance to *M.tb* infection and progression to TB.

## Do Genetic Variants of the Host Influence TB Drug Efficacy, Toxicity, and Resistance?

Host genetic mutations are not typically associated with the development of drug resistance, which is most commonly attributed to bacterial gene mutations under the selection pressure of prolonged drug exposure. However, genetic polymorphisms in host drug-metabolizing enzymes may render drugs less active or inactive, reduce drug levels that can promote bacterial mutation, or cause drug toxicity *via* increased formation of reactive metabolites (Motta et al., 2018). Such polymorphisms may contribute to differences in the incidence of anti-TB drug-induced hepatotoxicity (ATDH) between different populations. Among the anti-TB drugs, INH metabolism and disposition are strongly impacted by genetic factors. A few reports suggest a role for genetic polymorphisms with respect to rifampicin (RFP) metabolism, while less is known regarding the influence of genetic polymorphisms on the metabolism of other first- and second-line anti-TB drugs (Ramachandran and Swaminathan, 2012). Two major host enzymes involved in INH metabolism are NAT2 and cytochrome P450 family 2 subfamily E member 1 (CYP2E1). Frequent *NAT2* variants convey a rapid and slow acetylator phenotype, which inactivates INH as an anti-TB drug and results in substantial differences in drug exposure for the same dose. In addition, the main metabolite downstream of acetylation is acetylhydrazide, which is subsequently oxidized to toxic products. Since acetylhydrazide itself is further inactivated to diacetylhydrazide by NAT2, the relationship between NAT2 metabolizer status and liver toxicity results from the interplay of multiple pathways—an illustration that genetic effects can be complex even in a seemingly simple drug-effect relationship. A review by Klein et al. (2016) suggests that INH dose reduction in slow acetylators relative to rapid acetylators does not reduce anti-TB efficacy while likely reducing liver toxicity. Another recent study has reported an association between anti-TB drugs and *CYP2B6* polymorphisms and the risk of ATDH in a Chinese population (Wang et al., 2017). In their review of pharmacokinetics and pharmacogenetics of anti-TB drugs, Motta et al. (2018) suggest that increasing the RFP doses in individuals with SLCO1B1 loss of function alleles is a promising strategy—SLCO1B1 is a transporter of drugs into hepatic cells. More robust clinical prospective studies are needed to evaluate the contribution of these polymorphisms in the occurrence of liver toxicity during anti-TB treatment. Thus, we feel that there is a need to incorporate pharmacogenomics into clinical trials of TB to understand the factors impacting therapeutic success and occurrence of adverse drug effects.

## Discussion and Future Directions

Inter-individual variability in human immune responses to TB and other infectious diseases will continue to create a challenge as improvements in drugs and vaccines are discovered and implemented. Although currently viewed as provocative and difficult to achieve in infectious diseases, particularly in developing countries, we feel that the study and development of more personalized treatments and vaccines targeting specific immune characteristics of individuals, populations, or regions will become more important going forward. These more personalized approaches are likely to become commonplace as discoveries and innovations continue to be made in functional genomics and systems biology as applied to differential immune responses to disease. Currently, these innovations are being incorporated clinically in the fields of cancer, cardiovascular disorders, and brain diseases (Twilt, 2016).

The emergence and rapid dissemination of multi-drug, extremely drug, and totally drug-resistant strains *of M.tb* remains a severe global threat (Parida et al., 2015; Oppong et al., 2019); thus, development of new treatments and an effective vaccine(s) is paramount. We feel that it is also imperative to better understand the genetics underlying human variations in immune responses to TB, not only to aid in more personalized approaches to administration but also to advance host-directed or gene-targeted (e.g., CRISPR gene editing) therapeutic approaches that hold promise for overriding TB drug resistance.

Next-generation sequencing, increasing public availability of large-scale genomic and other “omic” data sets, and robust analytics need to be integrated into the diagnosis, treatment, and control of TB. Genomic data must be integrated with clinical and radiological data to compose complete individualized biological databases. Global challenges exist in differences between cultures, legal frameworks, economic conditions, and health priorities that have the potential to lead to a diversity of personalized medicine models throughout the globe, especially applicable to infectious diseases. To provide scientific evidence that will turn the concept and promise of personalized medicine into a reality for TB, the development of rigorous research programs, and both medical and non-medical multidisciplinary teams are required. If the correct policies and technologies are used, personalized medicine has the potential to improve treatment outcomes and decrease the transmission of TB for a greater proportion of the population.

In focusing on genetic studies, we note that the majority of SNPs associated with TB susceptibility have been found in intergenic regions of the genome, or non-coding regions of candidate genes (introns and UTRs), suggesting that they are regulatory genes (Azad et al., 2012; Uren et al., 2017). Recently, it has become possible to predict the function of these non-coding genetic variations by using software such as RegulomeDB or Variant Effect Predictor (Uren et al., 2017). The potential to identify the function of regulatory genes will allow for future studies to target those that lead to immune function changes and can be compensated with corrective drugs or supplements. These methods have already been used in the search for cures to diseases such as late-onset Alzheimer's, cardiovascular disease, and aging, suggesting that they could be used in the study of infectious diseases like TB as well (Rosenthal et al., 2014; Haider and Faisal, 2015; Bastami et al., 2016; Cavalli et al., 2016). Lastly, we propose that future clinical studies of TB therapy must link pharmacogenomics data with TB treatment outcomes.

We acknowledge that the concepts and ideas put forth in this PERSPECTIVE piece represent a leap for infectious diseases and TB in particular, and several significant roadblocks exist at the current time, including cost, technological tools, the feasibility of global data access and integration, etc. However, infectious diseases contribute substantially to global mortality and morbidity (Dye, 2014), and projections are for the impact of these diseases to increase (Holmes et al., 2017). TB alone has thus far killed over 1.4 billion people over human history, far greater than any other single infectious disease, and prevalence of drug resistance, toxicity of complicated drug regimens, and the absence of an effective vaccine that consistently generates protection in the majority of recipients portends continued challenges for TB control in the foreseeable future. These serious issues make it necessary that we continue to harness and embrace new intervention approaches.

## Author Contributions

All authors listed have made a substantial, direct and intellectual contribution to the work, and approved it for publication.

### Conflict of Interest

The authors declare that the research was conducted in the absence of any commercial or financial relationships that could be construed as a potential conflict of interest.
